# Unconventional modes of peptide–HLA-I presentation change the rules of TCR engagement

**DOI:** 10.1093/discim/kyac001

**Published:** 2022-05-04

**Authors:** Jade R Hopkins, Bruce J MacLachlan, Stephen Harper, Andrew K Sewell, David K Cole

**Affiliations:** Division of Infection and Immunity and Systems Immunity Research Institute, Cardiff University School of Medicine, Heath Park, Cardiff, UK; Division of Infection and Immunity and Systems Immunity Research Institute, Cardiff University School of Medicine, Heath Park, Cardiff, UK; Immunocore, Abingdon, UK; Division of Infection and Immunity and Systems Immunity Research Institute, Cardiff University School of Medicine, Heath Park, Cardiff, UK; Division of Infection and Immunity and Systems Immunity Research Institute, Cardiff University School of Medicine, Heath Park, Cardiff, UK

**Keywords:** T cells, human leukocyte antigen (HLA), peptide presentation, antigen recognition, protein flexibility, computational simulations

## Abstract

The intracellular proteome of virtually every nucleated cell in the body is continuously presented at the cell surface *via* the human leukocyte antigen class I (HLA-I) antigen processing pathway. This pathway classically involves proteasomal degradation of intracellular proteins into short peptides that can be presented by HLA-I molecules for interrogation by T-cell receptors (TCRs) expressed on the surface of CD8^+^ T cells. During the initiation of a T-cell immune response, the TCR acts as the T cell’s primary sensor, using flexible loops to mould around the surface of the pHLA-I molecule to identify foreign or dysregulated antigens. Recent findings demonstrate that pHLA-I molecules can also be highly flexible and dynamic, altering their shape according to minor polymorphisms between different HLA-I alleles, or interactions with different peptides. These flexible presentation modes have important biological consequences that can, for example, explain why some HLA-I alleles offer greater protection against HIV, or why some cancer vaccine approaches have been ineffective. This review explores how these recent findings redefine the rules for peptide presentation by HLA-I molecules and extend our understanding of the molecular mechanisms that govern TCR-mediated antigen discrimination.

## Introduction

Human leukocyte antigens (HLAs) are a family of genetically diverse cell surface-expressed glycoproteins, part of the major histocompatibility complex family that were first discovered in mice in 1936 [1, 2]. Their primary role is to display peptide fragments, generated by the cleavage of cytosolic proteins, at the cell surface [3, 4], allowing αβ T cells to interrogate the proteome for foreign or dysregulated proteins [5, 6]. HLA molecules are divided into two broad families: HLA class I (HLA-I) and HLA class II (HLA-II). These two main HLA classes are further divided into three subfamilies based on distinct genetic loci in humans: *HLA-A, HLA-B*, and *HLA-C* for HLA-I, and *HLA-DR, HLA-DQ,* and *HLA-DP* for HLA-II. HLA-I is expressed on almost all nucleated cells, primarily presenting peptides from endogenous sources to CD8^+^ T cells [7]. In contrast, HLA-II expression is generally restricted to antigen presenting cells and thymic epithelial cells [8], but can be induced in some other cell types by cytokines such as IFN-γ [9,10], and mainly presents peptides from exogenous sources to CD4^+^ T cells [7]. These classical HLA molecules are structurally related to a larger family of non-polymorphic antigen presenting molecules that can have distinct immunological functions and present chemically diverse antigens. These include the non-classical HLA-1b molecules (HLA-E, -F, -G and -H) which present peptide antigens [11], the cluster of differentiation (CD) 1 A, B, C, and D molecules which present lipid antigens, and the major histocompatibility complex, class I-related (MR1) protein which presents small metabolites [12]. Thus, T cells have the ability to monitor cellular dysregulation and foreign antigens *via* an array of complementary mechanisms.

Recognition of peptide–HLA (pHLA) complexes is mediated by the cell surface-expressed αβ T-cell receptor (TCR), first described in 1974 [13]. Functional TCR–pHLA interactions (defined by an interaction of sufficient strength/duration to initiate a T-cell signalling cascade [14]) can result in the activation of; CD8^+^ killer T cells (*via* TCR–pHLA-I interactions) to directly destroy aberrant target cells, or CD4^+^ helper T cells (*via* TCR–pHLA-II interactions) that can have a broad array of functional outcomes, from releasing proinflammatory cytokines and activating B cells to regulating immune activity [5, 6]. The TCR–pHLA interaction is also central to the process of maturation and selection that thymocytes (i.e. progenitor T cells) undergo whilst developing in the thymus [15, 16].

**Figure 1:**
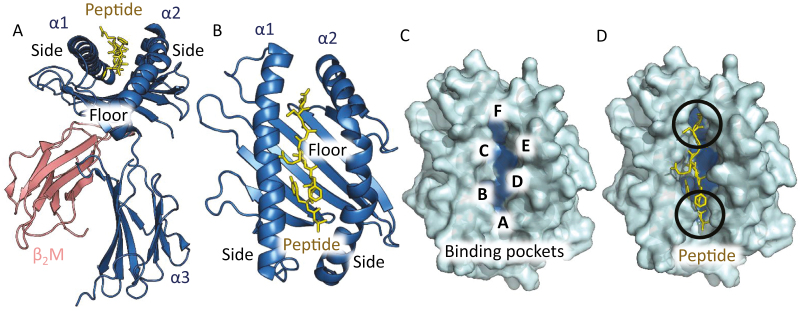
Overview of the pHLA-I complex and the peptide-binding groove. (**A**) Side view of the HLA-I α1, α2 and α3 domains (blue cartoon) with β_2_m (pink cartoon). The HLA-I α1, α2 domains form the peptide-binding groove (peptide shown as yellow sticks). (**B**) Top view of the α1 and α2 domains (blue cartoon) showing the seven-stranded β-pleated sheets which form the floor of the binding groove. (**C**) Top view of the α1 and α2 domains as surface representation (cyan). The binding groove is labelled according to the different binding pockets (A-F) that interact with the peptide. (**D**) Top view of the α1 and α2 domains as surface representation (cyan) showing the peptide (yellow sticks) with the buried anchor residues in black circles.

HLAs are encoded by one of the most diverse set of alleles in the human genome [2]. The HLA-I loci contains >400 genes and spans 4 Mb on chromosome 6 [17] with each HLA-I subfamily containing many thousands of alleles [18]. Current figures indicate there are >7000 alleles for HLA-A, nearly 9000 alleles for HLA-B and >7000 alleles for HLA-C [19]. HLA-I is a heterodimer comprising of a heavy chain, which includes the α1, α2 and α3 domains, non-covalently associated with the invariant beta-2-microglobin (β_2_m) chain [15,20] (Fig. 1A). The α1 and α2 helices form the peptide-binding groove, and a seven-stranded β-sheet that forms the floor of the binding groove (Fig. 1B). Within the peptide-binding groove there are six pockets, A-F (Fig. 1C), [21] that are responsible for most of the contacts with the peptide (Fig. 1D) [22]. Most HLA-I–restricted peptides conform to a conserved set of rules in which the peptide is presented in an extended conformation, tethered at its N and C termini by extensive contacts between the HLA groove and the peptide backbone. The peptide is oriented such that side chains from the second and final residues, known as the primary anchors, typically interact with the HLA-I B- and F-pockets, respectively (Fig. 1C and D). The HLA-binding pockets contain the highest level of sequence diversity between different HLA-I alleles, altering their preference for different amino acid types in the peptide and, as such, influencing the peptide repertoire compatible with binding to individual HLA-I molecules [18]. By presenting distinct peptide repertoires on different alleles, selective pressure that limits presentation of a pathogen on one allele will have limited impact in the population as individuals exposed to the same pathogen are likely able to present many other peptides on other alleles.

The shortest peptide that can reach between the B- and F-pockets is an 8-mer, resulting in a relatively “flat” conformation with the peptide stretched out along the length of the HLA-I groove [23]. Because the HLA-I binding groove is closed at each end, the central residues in peptides longer than eight amino acids are usually forced upwards, creating a central solvent exposed bulge, typically providing the main antigenic feature available for TCR interrogation. This closed-ended binding groove also limits the length of most peptides that can be accommodated by the MHC-I groove to 10–11 amino acids. However, several recent studies have identified multiple ways in which peptides can break these “rules of engagement,” redefining and extending our understanding of the molecular interactions that govern T-cell antigen recognition (summarized in Table 1). Here, we review recent findings that reveal how protein dynamics and flexibility at the peptide–HLA-I interface play an important role in shaping the nature of T-cell antigen recognition.

**Table 1:** Summary of pHLA-I ‘rule breakers’

Class of pHLA-I ‘rule breaking’	Definition	Refs
Super-bulged peptides	HLA-I can present peptides >11 residues for TCR recognition by generating large solvent exposed ‘super-bulges’ in the centre of the peptide whilst maintaining canonical peptide anchor residues.	[24–32]
Extending beyond the groove	Some peptides have been shown to extend beyond the boundaries of the ‘closed’ HLA-I binding groove by ‘kinking’ out at the N or C terminus, or by opening the groove around the F-pocket.	[33–39]
Heteroclitic peptides alter TCR binding	Peptide with modifications to buried anchor residues that modulate TCR recognition.	[40–47]
Peptide conformational switching	The same peptide sequence can be presented in very different conformations by different HLA-I alleles based on micro-polymorphisms in the binding pockets. This can lead to disease associations with different HLA-I alleles.	[48]
Peptides tune HLA-I flexibility	HLA-I protein motions are tuned/guided by the peptide. These differences in flexibility can directly affect TCR recognition.	[49–53]
Peptide-mediated changes in HLA-I	Peptides can alter the surface of the HLA-I binding groove, modifying antigenic features that impact TCR recognition.	[54–58]
Drugs modulation of the groove	It has been shown that drugs can make specific interactions with HLA-I binding pockets, altering the chemical nature of the pockets, and resulting in the presentation of different peptide repertoires that can induce drug hypersensitivity.	[59–63]
Peptides tune HLA-I flexibility	HLA-I protein motions are tuned/guided by the peptide. These differences in flexibility can directly affect TCR recognition.	[49–53]
TCR-induced alterations in peptide presentation	TCRs that capture the same pHLA in different conformations, or induce structural alterations when binding.	[25,27,64–68]

## Protein dynamics and flexibility guide the HLA-I peptide editing pathway

The peptides presented by HLA-I molecules are largely derived from cytosolic proteins that have been degraded by proteinases or, after ubiquitination, by the proteasome, or immunoproteasome following inflammation/cellular stress [69–72]. Thus, the adaptive immune system is dependent on the constant turnover of the proteome [73]. Inducible domains in the immunoproteasome fine-tune the specificity of the catalytic units to generate peptides of an appropriate size and specificity for HLA-I [74]. Peptides cleaved by the respective proteasomes may not be an optimal length for HLA-I presentation (8–11mers). Consequently, they can be cleaved further by cytosolic aminopeptidases such as leucine aminopeptidase, puromycin-sensitive aminopeptidase, bleomycin hydrolase, and tripeptidyl peptidase-II [73]. Following degradation, some peptide fragments are translocated into the endoplasmic reticulum (ER) by the transporter associated with antigen processing (TAP) protein complexes [69]. Peptides remain tethered to TAP after translocation to the lumen of the ER, where they can be further cleaved by the ER aminopeptidase associated with antigen processing, ERAP1/2, trimming them down after insertion into the HLA-I binding pocket [73, 75–77]. Free HLA-I heavy chains in the ER lumen are associated with the chaperone calnexin, which promotes the association of the HLA-I heavy chain with β_2_m [69]. Upon this association, calnexin dissociates in favour of another chaperone protein with a similar function, calreticulin [78, 79]. The newly generated “empty” HLA-I/β_2_m complexes are then recruited to TAP by tapasin, which also ensures that high-affinity peptides are preferentially presented [73, 80].

Recently, two groups have published structural data that have revealed in greater detail the dynamic and flexible nature of HLA-I in determining the outcome of the peptide editing described above. The crystal structures of HLA-I in complex with TAP binding-protein related (TAPBPR), a closely related homolog of tapasin, show how TAPBPR distorts the peptide-binding groove of the HLA-I in order to select for high-affinity peptides [81, 82]. TAPBPR consists of an N-terminal domain made up of seven-stranded β barrel and immunoglobin (Ig)-like (V type) folds, and a C-terminal IgC1 domain. The N-terminal domain has a large concave surface that encompasses the α2-1 helix region of the HLA-I heavy chain in a glove-like manner [81, 82]. In TAPBPR, a loop structure has been shown to modulate the specificity of the chaperone by selecting for peptides of optimal affinity. One group proposed that the loop acts as a scoop, inserting into the F-pocket and acting to stabilize HLA-I and ensuring competition with only high-affinity peptides [83]. A second group showed that removal of this loop leads to weaker binding of peptide to HLA-I and proposed an alternate hypothesis by which this loop sits above the peptide trapping transient pHLA-I interactions [84].

Comparison of free and TAPBPR-bound forms of HLA-I also showed dramatic movement in the α3 domain which shifts by 6.4 Å compared to the TAPBPR-unbound state [82]. Significant conformational alterations also occur in the F-binding pocket, which normally accommodates the C-terminal residue of the peptide [22,82]. F-pocket distortion generates knock-on effects to the distal A- and B-pockets which coordinate the N terminus of the bound peptide [81,82]. Thus, binding to an optimal peptide induces a conformational change in the HLA-I molecules, and as a result it adopts a closed conformation and dissociates from tapasin, enabling translocation of the newly formed peptide–HLA-I complex to the cell surface *via* the exocytotic pathway [73]. Thus, the intrinsic flexibility of the HLA-I platform enables selection of the peptide repertoire for each HLA-I allele for presentation at the cell surface, and interrogation by TCRs on circulating CD8 T cells.

## Peptides that break the rules

### pHLA-I flexibility enables unconventional peptide presentation

The HLA-I binding groove is ideally suited to bind relatively short peptides (8–11mers) in an extended form. However, it has been shown that longer peptides can also be accommodated in the HLA-I binding groove [24–31], raising questions as to their presentation mode and whether they are still functional T-cell epitopes.

**Figure 2:**
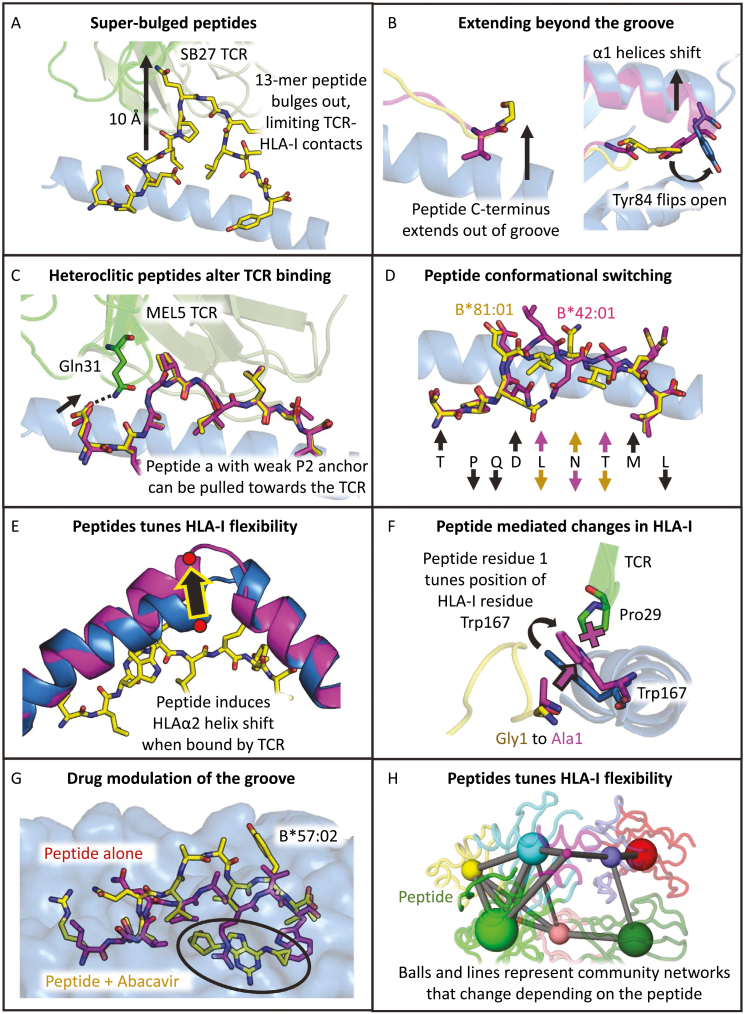
Different modes of pHLA-I ‘rule breaking’. (**A**) The HLA-B*35:08-restricted 13-mer (LPEPLPQGQLTAY) peptide (yellow sticks) bulging out of the HLA-I groove (blue cartoon) limiting contacts between the SB27 TCR (green cartoon) and the HLA-I helices. (**B**) LEFT: C-terminal residue of the MLLSVPLLLG peptide (yellow sticks) extending out of the groove. C-terminal peptide residue usually points down into the groove as a primary anchor (pink sticks). RIGHT: 10-mer LYLVCGERGF peptide (yellow sticks) extends out of the side of the binding groove, mediated by residue Tyr84 (blue sticks) flipping into an open conformation and inducing a widening of the binding groove (black arrows). (**C**) The MEL5 TCR (green cartoon) ‘pulls’ the E**A**AGIGILTV peptide (yellow sticks) away from the HLA-I binding groove (shift shown by black dotted line and arrow) making optimised contacts with TCR residue Gln31 (green sticks) compared to the heteroclitic anchor residue optimised version of the peptide (E**L**AGIGILTV shown as pink sticks). (**D**) The TL9 (TPQDLNTML) peptide adopts unique conformations when presented by HLA-B*81:01 (yellow sticks) and HLA-B*41:01 (pink sticks). In HLA-B*81:01, residues Leu5 and The7 point down and Asn6 points up (indicated by coloured arrows above and below peptide sequence). In HLA-B*41:01, these residues are inverted, with residues Leu5 and The7 pointing up and Asn6 pointing down. These changes are attributed to micro-polymorphisms between HLA-B*81:01 and HLA-B*41:01. (**E**) The Tel1p (MLWGYLQYV) peptide (yellow sticks) induces a conformational change in the HLAα2 helix (blue cartoon to pink cartoon and labelled with a black arrow) upon A6 TCR binding (TCR not shown). (**F**) GVY01 TCR (green cartoon) residue Pro29 (green sticks) can detect alterations in the MAGE-A4 (GVYDGREHTV) peptide at the first residue (yellow and pink sticks) due a conformational change (black arrow) in HLA-I residue Trp167 (blue and pink sticks). (**G**) Abacavir (yellow sticks and circled in black) can bind into the F-pocket HLA-B*57:02 (blue surface), altering the chemical nature of the binding pocket. This blocks the binding of natural HLA-B*57:02-restricted peptides (pink sticks) due to steric repulsion, altering the nature of the presented peptide repertoire (peptide + abacavir shown in yellow sticks). (**H**) Representation of the correlated network of motions, found to be dependent on peptide sequence, analysed by molecular dynamics simulation. Networked communities shown as coloured spheres, with larger spheres indicating a greater number of residues within the community. Lines represent communication pathways between the nodes, with thicker lines indicating a greater degree of correlation between the two communities. It was found that different peptides, bound to the same HLA-I molecule, could modulate these networks (‘the tail wags the dog’) with implications for antigen processing, co-receptor binding, and TCR recognition.

To date, two different modes of accommodating these extra residues have been reported. The first mode involves longer peptides forming extended central bulges to accommodate the extra residues, also known as “super-bulged” peptides. In general, longer peptides that are anchored at their termini have increased flexibility in the central residues and a greater prevalence of glycine residues (as with the two examples below highlighted in italics) facilitating their increased flexibility and ability to adopt the necessary bulged conformation [85]. Longer peptides being accommodated in the groove is best exemplified in the crystal structures of HLA-A*02:01 in complex with two 15-mer peptides (F**L**NKDLEVD*G*HFVT**M** and A**L**QDA*G*DSSRKEYF**I**), showing that both adopted a “super-bulged” conformation, anchoring with their leucine and methionine/isoleucine residues (bold in the sequences shown above) in the B- and F-pockets, respectively [32]. Another study, focussed on an HLA-B*35:08-restricted 13-mer from the Epstein–Barr virus (LPEPLPQ*G*QLTAY) [25,30], revealed the molecular consequences of “super-bulged” peptides on TCR recognition. In this example, the central bulged region of the peptide protruded out of the antigen-binding cleft and reached ~10 Å further into the solvent than an HLA-B*35:08-restricted 8-mer (Fig. 2A). Structural analysis demonstrated that the SB27 TCR was “perched” on top of the central bulge, limiting contacts with the HLA-I surface and enabling the TCR to adopt two different binding orientations [26].

The second mode by which HLA-I accommodates longer peptides involves peptides extending beyond the conventional boundaries of the closed HLA-I binding groove, often mediated by amino acids that are theoretically incompatible with the B- and F-pockets of certain HLA alleles [86]. Several structural analyses have shown that these peptides can have only one termini properly anchored, with either N- or C-terminal extensions occurring *via* extending out from either the A- or F-pocket, respectively. For example, in 1994, Collins *et al.* used X-ray diffraction to determine the structure of HLA-A*02:01 in complex with the MLLSVPLLLG peptide and demonstrated that, although the first nine residues of the peptide bound to HLA-A*02:01 conventionally, the pattern of hydrogen bonds formed at the C terminus was abnormal [33]. Typically, four hydrogen bonds exist between the HLA-I and the terminal carboxylate oxygens, three of which are formed by conserved HLA-I residues (Thr143, Lys146, and Tyr84), and the fourth from a non-conserved residue (Thr80 in the case of HLA-A*02:01). In the C-terminally extended example, only one of these H bonds was preserved (Thr143). Two of the four HLA-I residues normally involved with this H-bond network had undergone a conformational change. Tyr84 had rotated away from the peptide (Ty84 flip), and Lys146, which typically caps the F-pocket burying the peptide, had been raised out of the binding groove. Together, these alterations created an opening through which the peptide could extend [33] (Fig. 2B). Similarly, a 10-mer peptide from the murine insulin peptide_15-24_ (LYLVCGERGF), in complex with H-2K^d^, could be anchored at position 9, with residue 10 extending out of the groove (Fig. 2B). The extension at residue 10 was again enabled by a Tyr84 flip. Additionally, McMurtrey *et al.* recently showed that several HLA-A*02:01-restricted *Toxoplasma gondii*-derived epitopes exhibited canonical N terminal binding but could extend anywhere from 1 to 30 amino acids at the C terminus [37]. Finally, Pymm *et al.* analysed four different HLA-B*57:01-restricted peptides, including the HIV Gag epitope TW10_240-249_ (TSTLQEQIGW) [34]. In these structures, peptide residue P3, rather than P2, acted as the primary anchor residue, enabling Ser2 to rotate 180° in the A-pocket of the HLA-I molecule, positioning Thr1 close to position 62, a glycine in this HLA-I allele. This unusual backbone geometry of placing the −1 residue adjacent to position 62 is shared in other crystal structures that have an N-terminally extended peptide. In HLA-B*08:01 presenting peptide variants, the A-pocket is opened up *via* a side-chain rotation in Arg62 which, in analogous manner to Tyr84 or Lys146 in the F-pocket, rearranges to accommodate the extended peptides [35, 36].

## Altered peptide–HLA-I interactions modify antigenic features

### Heteroclitic peptide modifications alter peptide presentation

As discussed above, allele-specific peptide repertoires are driven by polymorphisms in the HLA-I binding pocket, most critically the downward facing “primary anchor” residues which generally bind to the HLA-I B- and F-pockets. [22, 87, 88]. However, immunogenic peptides can still be presented even when these residues are suboptimal for binding to these HLA-pockets. Because the anchor residues are buried within the HLA-I molecule and not exposed to the TCR, it was assumed that introducing optimal anchor residues to create “heteroclitic” peptides would improve pHLA-I stability and immunogenicity of cancer vaccines without altering TCR recognition. However, we and others have demonstrated that alterations in the HLA-I anchor residues can have unanticipated knock-on effects on TCR specificity [40–46, 89]. Thus, the use of heteroclitic peptides (defined as peptides with altered anchor residues to optimize pHLA stability) as vaccines may select T cells that do not recognize the intended natural target, resulting in poor function *in vivo.* Importantly, the ability of changes in HLA-facing “buried” primary anchor residues to alter interactions with TCRs raises questions about the effectiveness of using heteroclitic peptides in vaccine trials without detailed structural/mechanistic understanding on how these peptides are presented to T cells [64, 90–94]. For example, the weaker anchor residue Ala2 in the HLA-A*02:01-restricted MART-1/Melan-A_25-36_ peptide (E**A**AGIGILTV), which is overexpressed in melanoma, allowed the MEL5 TCR to “pull” the N terminus of the peptide away from the HLA-I groove, facilitating new contacts that were not able to form when the anchor residue was optimised in the heteroclitic E**L**AGIGILTV peptide [41, 95] (Fig. 2C). The enhanced binding was reflected by the greater sensitivity of the MEL5 CD8^+^ T cell clone to recognize HLA-A*02:01-E**A**AGIGILTV, compared to its heteroclitic counterpart, HLA-A*02:01-E**L**AGIGILTV. These findings were reflected by clinical trials demonstrating that vaccination using the native E**A**AGIGILTV peptide-induced stronger tumour reactivity than using the heteroclitic version of the peptide (E**L**AGIGILTV) [92]. Another recent study demonstrated that different TCRs could distinguish between the wild-type HLA-A*02:01-restricted gp100_209-217_ (ITDQVPFSV) peptide and its anchor residue modified heteroclitic variant (I**M**DQVPFSV), with some TCRs binding more tightly and some more strongly, with up to 40-fold differences in affinity. Intriguingly, there appeared to be no structural explanation for the differences in affinity, with the changes attributed to dynamic allostery, whereby substitutions to the anchor residue at position 2 in the peptide caused long-range effects in peptide residues distal from the mutation site that altered TCR binding [89].

To further understand how peptide positions that do not directly contact the TCR impact T-cell specificity, heteroclitic peptide variants with substitutions at non-anchor positions have been studied using structural, cellular, and biophysical approaches. For instance, we demonstrated that the GP100 TCR that recognizes the HLA-A*02:01-restricted gp100_280-288_ (YLEPGPVTV) peptide was highly sensitive to mutation of peptide residue Glu3 to Ala3, despite this residue making no contact with the TCR [43]. Further structural analysis of HLA-A*0201-YL**A**PGPVTV demonstrated that substituting position 3 for alanine resulted in a molecular switch which not only affected the residue in question, but also had knock-on effects that were transmitted to other TCR contact residues [43]. Finally, we demonstrated that the ILA1 TCR, which recognizes an HLA-A*02:01-restricted human telomerase reverse transcriptase_540-548_ peptide (ILAKFLHWL), can detect changes in the peptide at residue 8, away from the canonical central bulge in the peptide [96, 97]. Structural analysis demonstrated that, although the ILA1 TCR did not contact peptide residue 8, substitutions at this position resulted in an alteration in the overall conformation of the peptide backbone [46].

### TCR-induced alterations in peptide presentation

As well as the TCR being able to indirectly sense changes in distal regions of the peptide, the same pHLA-I complex can be captured in distinct conformations depending on interactions with the TCR. For instance, structural information has emerged demonstrating that an 11-residue peptide (EPLPQGQLTAY) from the BZLF1 antigen of the Epstein–Barr virus presented by HLA-B*35:01 is “bulldozed” into a flatter conformation upon ligation with the ELS4 TCR, with the side chains of two glutamine residues (at positions 5 and 7) undergoing a significant movement and facilitating new contacts between the TCR and the peptide [25]. The ability of the same pHLA to be recognised in distinct conformations by different TCRs has also recently been described for the HLA-A*02:01-restricted cancer testis antigen, NY-ESO-1_157–165_ (SLLMWITQC), where the central Met4 and Trp5 peptide residues form a dominant antigenic feature termed the MW-peg [65]. In more than 15 crystal structures, including with different TCRs and multiple heteroclitic peptides [64, 66, 67], the MW-peg was the dominant antigenic feature and always protruded from the HLA-I surface, such that recognition of this motif shapes the TCR repertoire [27,64–68]. Thus, it was surprising to identify a TCR–pHLA-I complex in which the central residues of the HLA-A*02:01-NY-ESO-1_157–165_ peptide (SLLMWITQC) were flipped, so that the Trp5 residue of the MW-peg was buried, with the next residue in the peptide, Ile6, which typically acts as a secondary anchor, pointing away from the HLA surface and making an extensive TCR interface [65]. In, perhaps, the most extreme example of TCR-induced pHLA alterations, Riley *et al.* demonstrated that the clinically relevant HLA-A*02:01-MART-1_26-35_ (EAAGIGILTV) reactive DMF5 TCR could cross-react with a structurally and biochemically distinct HLA-A*02:01-restricted peptide, MMWDRGLGMM [47]. Structural analysis demonstrated that, upon DMF5 TCR binding, the MMWDRGLGMM peptide underwent a gross structural rearrangement, resulting in a peptide register shift in which Met9 replaced Met10 as the C-terminal peptide anchor position in the HLA F-pocket, leading to the peptide C-termini extending out of the groove. This large conformational change in the peptide was accompanied by a shift in the position of the HLAα2 helix, resulting in the decamer peptide being presented more like a nonomer peptide in the complex. Together, these findings demonstrated that the TCR and peptide–HLA-I are not structurally fixed but can rearrange upon recognition of their target *via* induced fit, or by conformational selection of different low energy conformations. The implication for these observations is that a single pHLA-I molecule might represent several distinct epitopes that can be captured by different TCRs, making predictions of TCR specificity very challenging by considering peptide sequences alone.

### HLA-I polymorphisms can alter peptide presentation

There are many examples in which the HLA-I landscape in an individual can determine susceptibility to infectious diseases or autoimmune conditions [98,99]. For example, it has been shown that T-cell responses to the dominant HLA-B*07 epitope (TPQDLNTML) from the Gag protein are markedly different between individuals with HLA-B*07 alleles differing by only a single amino acid (known as HLA micro-polymorphisms) and these differences can drive susceptibility, or protection, against different clades of HIV [48].

To better understand the molecular mechanisms driving differences on disease outcome, we investigated the molecular basis for HLA-B*07-linked divergent HIV protection. By solving the structure of TPQDLNTML presented by the HLA-B*07 superfamily alleles HLA-B*42:01 and HLA-B*81:01, we demonstrated that single amino acid polymorphisms between otherwise identical HLA-I molecules could cause a conformational switch in the peptide (Fig. 2D) [48]. The alteration in the peptide presentation mode between the two alleles was also consistent with the emergence of differing escape mutants in individuals expressing different HLA-Is [48]. Thus, HLA-I polymorphisms generated distinct structural epitopes, even when presenting identical peptide sequences. This HLA micro-polymorphism-generated plasticity has important implications for TCR recognition and the role of HLA-type and association with disease outcome that was hitherto unappreciated.

### Peptide-mediated modulation of antigenic features

As well as HLA-I polymorphisms indirectly influencing antigenic features in the peptide, several studies have demonstrated that changes in peptide sequence can indirectly alter the conformation of the HLA-I binding groove, modulating TCR recognition. For example, we demonstrated that the human A6 TCR can recognize both the HTLV-1 Tax peptide (LLFGYPVYV) and the *Saccharomyces cerevisiae* peptide Tel1p (MLWGYLQYV) bound to HLA-A*02:01. LLFGYPVYV and MLWGYLQYV have a similar sequence and have superimposable structures when bound to HLA-A*02:01 [54]. However, when in complex with the A6 TCR, a substantial structural change was observed in the HLA-A*02:01-Te1p complex, in which the HLA α2 helix (Ala150-Val152) underwent a conformational switch that shortened the short arm of the helix and extended the long arm (Fig. 2E). The conformational switch in the HLA α2 helix resulted in Ala150 shifting to form the N-terminus of the long helix and knock-on effects were observed along the HLA α2 helix which was displaced away from the peptide.

Further evidence for HLA-I flexibility has emerged from studies investigating the A6 TCR recognition of a self-antigen from the human neuronal protein (HuD) [55, 56], thought to play a role in the neurologic disorder human T-cell leukaemia virus-1–associated myelopathy/tropical spastic paraparesis (HAM/TSP). Borbulevych *et al*. solved the structure of the A6-HLA-A*02:01-HuD complex and demonstrated that it was a “close but imperfect” structural mimic of HLA-A*02:01-Tax. Interestingly, the TCR engaged the self-ligand in the same manner as it engaged the Tax ligand [57]. However, conformational changes were observed in the HLA-I helices in the A6-HLA-A*02:01-HuD that did not occur in the A6-HLA-A*02:01-Tax complex, leading to an observed weaker binding affinity of the A6 TCR for HLA-A*02:01-HuD than for HLA-A*02:01-Tax (140 and 2 μM, respectively) [57].

Finally, we recently uncovered a novel mechanism of peptide recognition, mediated by an indirect interaction between the GVY01 TCR and peptide residue 1 in the MAGE-A4 (GVYDGREHTV) peptide *via* a molecular gateway in HLA-I residue Trp167 [58]. We found that, depending on the size and chemical nature of the amino acid side chain in the A-pocket, Trp167 underwent a conformational change that could completely abrogate GVY01 TCR binding (Fig. 2F). We also identified other published systems where this mechanism was apparent, implying a common binding mechanism that should be considered when studying TCR-mediated antigen recognition. Thus, the HLA-I surface can act as an extension of the peptide in instances where HLA-I conformation is the direct consequence of the peptide-mediated conformational change.

### HLA-I–associated drug hypersensitivity

In addition to susceptibility to infection, an increasing number of adverse immunological reactions to drugs have been associated with HLA-I polymorphisms, including abacavir hypersensitivity syndrome (AHS) [59–63]. Abacavir is a common treatment for HIV-1 infections, and AHS occurs exclusively in HLA-B*57:01-positive individuals [63]. It does not occur in individuals expressing closely related natural allotypes HLA-B*57:02, HLA-B*57:03 and HLA-B*58:01 despite there being as few as two amino acid differences from HLA-B*57:01 [100]. Structural evidence later demonstrated that abacavir was able to bind to the antigen-binding cleft of HLA-B*57:01, interacting primarily with the F-pocket, to which the carboxy-terminal tryptophan of a peptide usually binds (Fig. 2G). As such, abacavir altered the conformation and chemistry of the binding pocket, and consequently the repertoire of endogenous peptides that HLA-B*57:01 was able to bind and present at the cell surface. Altered binding of self-peptides resulted in a marked alteration in ‘immunological self’ which activated systemic abacavir-specific T-cell responses and manifested in patients as AHS. These findings demonstrate the importance of considering HLA-I polymorphisms as a factor during the evolution of pharmacogenetics and demonstrates how the peptide self-repertoire can be completely altered by drugs that interact with the HLA-I binding groove [59].

### Dynamic investigations of TCR–pHLA-I interactions reveal the role of protein flexibility during antigen engagement

Because the current understanding of the TCR–pHLA interaction has been dominated by crystallographic studies, investigations of TCR–pHLA flexibility have largely been inferred by comparing TCR–pHLA complexes to the corresponding unbound molecules [49]. These studies have implicated the TCR as the main driver of flexibility during TCR–pHLA interactions [101]. However, recent evidence, generated using isotope-edited infrared spectroscopy, nuclear magnetic resonance (NMR), Förster resonance Energy transfer (FRET), and molecular dynamics (MD) stimulation, has offered new insights into the flexibility of the TCR–pHLA interaction, and in particular of the pHLA complex [49–53, 102]. For example, recent evidence has shown that the SB27 TCR can discriminate between HLA-B*35:08 and HLA-B*35:01, presenting the same 13-mer peptide (LPEPLPQGQLTAY) in an identical fashion [25,26]. MD stimulation revealed that the HLA-B*35:01-peptide complex exhibited greater flexibility than its HLA-B*35:08-peptide counterpart, despite differing by only a single amino acid, an arginine to leucine substitution at position 156 for HLA-B*35:08 and HLA-B*35:01, respectively [49]. Similar observations were made for the 9-mer peptide (GRFAAAIAK) bound to either HLA-B*27:05 or HLA-B*27:09 [103]. The two HLAs only differ by a single amino acid substitution, and have a virtually identical crystal structure [104], yet MD stimulation revealed greater flexibility in the peptide–HLA-B*27:09 complex, which was attributed to the single polymorphism [49].

MD simulation, combined with high-pressure/temperature perturbation experiments, have also been used to explore how differences in the bound peptide govern molecular flexibility in different peptide–HLA-I complexes [53]. It has been shown that HLA-I motions can be dependent on, and mediated by, small alterations in the bound peptide, suggesting that allosteric mechanisms might mediate HLA-I flexibility (i.e. “the tail wags the dog”) (Fig. 2H). Differences in protein dynamics for different peptide cargo manifested primarily in the HLA-I molecule (as opposed to the peptide) and were found in several regions known to be involved in antigen processing (*via* tapasin and TAPBPR interactions) and CD8 co-receptor interactions, suggesting a role for the peptide in modulating these interactions. These observations were consistent with another study in which molecular dynamic simulations were performed on over 50 unique nonamer peptides in complex with HLA-A*02:01 [102]. These analyses demonstrated that different peptides could influence distal regions of the HLA molecule, including the α3 domain, and even the non-covalently bound β2m chain. Both studies suggest that these peptide-dependent changes in HLA flexibility/dynamics might directly influence the peptide repertoire selected during the HLA-I antigen processing pathway, as well as directly influencing TCR-mediated antigen discrimination. Finally, alterations in the HLA-bound peptide that affect the flexibility of the HLA-I can also alter TCR affinity. For example, peptide-induced flexibility has been demonstrated in the context of the MART-1 melanoma peptide (AAGIGILTV), which can alter the flexibility of the α1 and α2 helices of the HLA-A*02:01 molecule. Modifying the anchor residue at position 2 from alanine to leucine improved peptide binding to HLA-I and enhanced the flexibility of the pHLA-I complex, as measured by MD stimulation, reducing TCR affinity [105].

The constellation of evidence described above sheds new light on the molecular mechanisms guiding protein dynamics in the pHLA-I, the major target for the TCR. Peptide–HLA flexibility is of particular significance for understanding the molecular mechanisms that govern T cell-mediated antigen recognition and has strong implications for the growing community of researchers and companies developing therapies that target pHLA-I.

## Concluding remarks

The flexibility and adaptability of the pHLA-I molecule is becoming ever more apparent, with the “rules” that govern TCR recognition of pHLA-I being constantly expanded. It is highly likely that these “unconventional” modes of peptide presentation by HLA-Is are relatively common, but not always captured by crystallographic analysis alone. Thus, it is vital to understand the dynamic characteristics of these molecules, and to take into consideration the contribution of allostery, protein dynamics, protein flexibility, and interconnected protein networks during peptide presentation by HLA-I. It is also important to appreciate that the intrinsically dynamic and flexible nature of HLA-I molecules strongly suggests that TCRs select for both peptide and HLA conformation during binding, with the evidence presented in this review supporting the notion that (almost) anything can happen during TCR recognition of pHLA-I. In summary, “unconventional” peptide presentation driven by dynamic interactions with HLA molecules and TCRs has obvious implications for understanding the nature of TCR specificity and cross-reactivity and might help to uncover disease associations with different HLA alleles.

## Data Availability

Not applicable.

## Abbreviations

AHS: abacavir hypersensitivity syndrome
ER: endoplasmic reticulum
FRET: Förster resonance energy transfer
HAM/TSP: neurologic disorder human T-cell leukaemia virus-1–associated myelopathy/tropical spastic paraparesis
HLA-I: HLA class I
HLA-II: HLA class II
HuD: human neuronal protein
Ig: immunoglobin
MD: molecular dynamics
NMR: nuclear magnetic resonance
pHLA: peptide-human leukocyte antigen
TAP: transporter associated with antigen processing
TCRs: T-cell receptors
β_2_m: β-2-microglobin
